# Crystal structure of akuammicine, an indole alkaloid from *Catharanthus roseus*

**DOI:** 10.1107/S2056989017014529

**Published:** 2017-10-20

**Authors:** Mahdi Yahyazadeh, Gerold Jerz, Dirk Selmar, Peter Winterhalter, Peter G. Jones

**Affiliations:** a Institut für Pflanzenbiologie, Technische Universität Braunschweig, Mendelssohnstrasse 4, 38106 Braunschweig, Germany; b Institut für Lebensmittelchemie, Technische Universität Braunschweig, Schleinitzstrasse 20, 38106 Braunschweig, Germany; c Institut für Anorganische und Analytische Chemie, Technische Universität Braunschweig, Hagenring 30, 38106 Braunschweig, Germany

**Keywords:** crystal structure, indole alkaloid, absolute configuration

## Abstract

The title compound, C_20_H_22_N_2_O_2_, an alkaloid isolated from the Madagascar periwinkle, crystallizes in *P*1 with two independent but closely similar mol­ecules in the unit cell. The mol­ecules are linked into pairs by two N—H⋯O=C hydrogen bonds. The absolute configuration was confirmed by anomalous dispersion effects as *S* at the 3 and 15 positions, and *R* at the 7 position.

## Abstract 2

The structure of akuammicine, an alkaloid isolated from the Madagascar periwinkle, was confirmed and its absolute configuration determined.

## Chemical context   

The Madagascar periwinkle or rosy periwinkle (*Catharanthus roseus* L. G. Don), a member of the family Apocynaceae, is one of the most intensively studied medicinal plants (Sottomayor *et al.*, 1998 ▸; Sreevalli *et al.*, 2004 ▸). Aerial parts of the plant contain between 0.2 and 1% of a mixture of more than 120 alkaloids (van Der Heijden *et al.*, 2004 ▸). The most abundant are the monomers such as catharanthine and vindoline (Renault *et al.*, 1999 ▸). The dimeric alkaloids that result from the joining of two compounds can display inter­esting pharmaceutical activities. Thus vinblastine and vincristine are used in the chemotherapy of leukemia and in the treatment of Hodgkin’s disease (Verma *et al.*, 2007 ▸). Additionally, ajmalicine, a monomeric indole alkaloid present in the root of *C. roseus*, is an anti­hypertensive alkaloid (Noble, 1990 ▸). In view of their medical and commercial value, the appropriate methods of extraction and purification have been well studied.
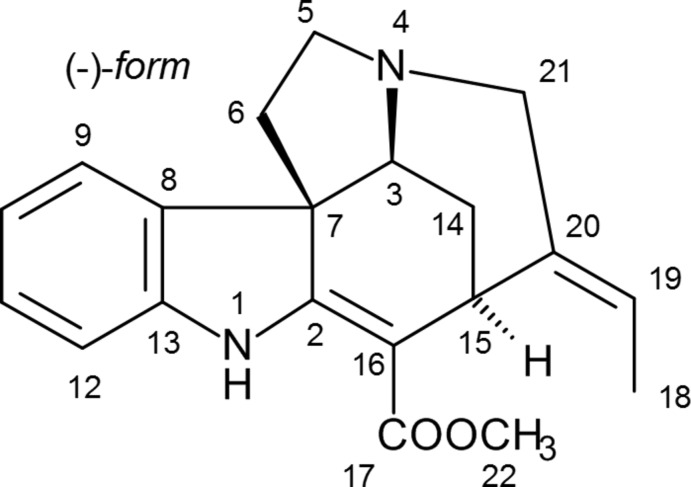


We have undertaken the X-ray crystal structure determination of the title compound in order to establish its absolute stereochemistry. One-dimensional (^1^H, ^13^C, DEPT135) and two-dimensional NMR (HSQC, HMBC, ^1^H/^1^H-COSY, ^1^H/^1^H-NOESY) experiments clearly assigned the proton and carbon resonances and are consistent with the constitution of akuammicine (Buckingham *et al.*, 2010 ▸). As the final purification step was performed with an alkaline solvent mixture, the NMR data of akuammicine correspond to the free base, and can be linked to the determined stereochemistry.

## Structural commentary   

**Figure 1:**
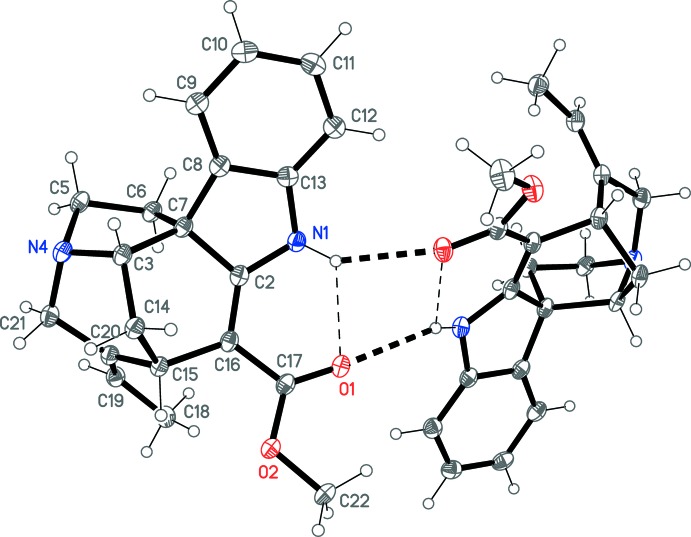
The structure of the title compound in the crystal. Ellipsoids represent 30% probability levels. Both independent mol­ecules are shown, but for clarity only one is labelled. The second mol­ecule has the same numbering but with primes. Dashed lines represent hydrogen bonds.

The title compound crystallizes in space group *P*1 with two independent mol­ecules (Fig. 1 ▸). The two mol­ecules are closely similar; a least-squares fit of all non-H atoms gives an r.m.s. deviation of 0.065 Å, whereby the largest deviation is 0.29 Å for the methyl carbon C18. The absolute configuration is established as *S* at C3 and C15 and *R* at C7. Intra­molecular classical N1—H01⋯O1 hydrogen bonds are observed (Table 1 ▸). The C18—H18*A*⋯O2 contacts in both mol­ecules may represent a significant intra­molecular inter­action.

The five-membered ring involving N4 displays an envelope conformation, with C5 lying outside the plane of the other four atoms. The cyclo­hexene ring is a ‘skew-boat’ or 1,3-diplanar form, with torsion angles of approximately zero about C3—C7 and C2=C16. Finally, the six-membered ring involving N4 shows a form inter­mediate between boat and skew-boat; the torsion angle about C15—C20 is approximately zero, but that about C3—N4 (which would also be zero for an ideal boat) is about 24°. See Table 2 ▸ for details.

## Supra­molecular features   

The two mol­ecules are connected by the two classical hydrogen bonds, N1—H01⋯O1′ and N1′—H01′⋯O1 (Fig. 1 ▸, Table 1 ▸), to form a dimeric assembly. The contacts C11—H11⋯*Cg*1(1 + *x*, −1 + *y*, *z*) and C11′—H11′⋯*Cg*2(−1 + *x*, 1 + *y*, *z*), where *Cg*1 is the mid-point of C19=C20 and *Cg*2 is the mid-point of C19′=C20′, may represent C—H⋯π inter­actions; the H⋯*Cg* distances are 2.74 and 2.72 Å, and the angles at H are 147 and 155°, respectively.

## Database survey   

The most similar natural product to have been investigated by X-ray structure analysis is probably isovoacangine (Soriano-García *et al.*, 1991 ▸), refcode KORZOG.

## Isolation and crystallization   

The title compound was isolated using a combination of high-performance countercurrent chromatography (HPCCC) (Ito, 2005 ▸), preparative C18 high-performance liquid chromatography (HPLC) and silica-gel column chromatography (Ito, 2005 ▸). Seedlings of F1 Titan Rose *Catharanthus roseus*, purchased from a commercial provider of pharmaceutical plants (Gärtnerei Volk GmbH Pflanzenhandel, Braunschweig, Germany), were planted and grown outside from June to July 2015 on a mixture of standard garden soil and sand (2:1). Aerial plant parts were harvested and lyophilized, and dried tissue material was then milled by a bead mill [Mixer Mill MM 200 (RETSCH, Haan, Germany) at a vibrational frequency of 25 Hz for 1 min]. The dried powder was immersed in water adjusted to pH 2 by tri­fluoro­acetic acid (TFA), homogenized by a T-25 digital ULTRA-TURRAX (IKA, Staufen, Germany) at maximum speed (25 000 rpm for 10 min) and shaken overnight for extraction. Plant particles were centrifuged off (30 min, 8000 rpm). The acidic extract was lyophilized and redissolved in 1 l chloro­form. A solution of NaOH was added (1 l, 200 m*M*) and the solutions were vigorously mixed for alkaloid extraction. The phases were centrifuged (8000 rpm, 15 min) and the chloro­form phase was dried for indole-alkaloid recovery.

The complex indole-alkaloid crude extract (700 mg) was injected into a semi-preparative HPCCC instrument (Spectrum, Dynamic Extractions Ltd, Gwent, UK) (Ito, 2005 ▸), a J-type centrifuge equipped with two coil bobbins (PTFE tubing, ID 1.6 mm, column volume 125 ml) operated with the biphasic solvent system water/*n*-hexane/*n*-butanol (2:1:1 *v*/*v*/*v*) using the ion-pair reagent TFA (5.0 ml l^−1^). The rotation velocity was set to 1600 rpm (240 *g* field), and the flow rate of the aqueous mobile phase (5.0 ml min^−1^) (head-to-tail mode) resulted in a stationary phase retention of 60% after system equilibration.

For metabolite profiling, aliquots of the recovered HPCCC fractions were injected in sequence into an ESI-ion trap MS/MS (HCT Ultra ETD II, Bruker Daltonics, Bremen, Germany) in a standard protocol described by Jerz *et al.* (2014 ▸) and the target alkaloid akuammicine was detected with [*M*+H]^+^ at *m*/*z* 323 in fractions 61 to 69 (elution volume 304–345 ml). The combined fractions were re-chromatographed by preparative HPLC (Wellchrom K-1001, Knauer Gerätebau Berlin, Germany) using a C18 column (Prontosil C18Aq, 25 × 250 mm) and an isocratic flow rate of 4.5 ml min^−1^ (aceto­nitrile:water, 60:40 with 1% TFA). Alkaloids were monitored using a UV detector (Wellchrom K-2600, Knauer Gerätebau, Berlin, Germany) at λ 254, 280 and 300 nm.

The 10 mg amber-coloured HPLC fraction was finally purified by SiO_2_ column chromatography (Merck, Darmstadt, Germany) using ethyl acetate/*n*-hexane/ethanol/25% aqueous ammonia (100/5/5/3) to yield pure akuammicine (1.2 mg), detected by thin-layer chromatography (TLC) (SiO_2_ 60 F254, Merck, Darmstadt, Germany) with this solvent system and sprayed with Dragendorff reagent (*R*_F_ value 0.25). LC–ESI–MS, measured in the positive ionization mode using a Prontosil C18-Aq column (250 × 2.0 mm, 5 µm, 100 Å, Knauer Gerätebau, Berlin, Germany), detected akuammicine, ESI–MS/MS (pos) [*M*+H]^+^: *m*/*z* 323, MS/MS 291 (100%).

Akuammicine crystals grew in tube fractions during slow evaporation of the solvents, and an appropriate colourless crystal was chosen for X-ray analysis.

^1^H NMR (FT 300, Bruker Biospin, Rheinstetten, Germany, 300 MHz, CDCl_3_), calibrated to tetramethylsilane (TMS), δ (p.p.m.): 8.97 (1H, *s*, NH-1), 7.39 (1H, *d*, *J* = 7.5 Hz, H-9), 7.24 (1H, *dt*, *J*_1_ = 8.0 and *J*_2_ = 0.8 Hz, H-11), 6.98 (1H, *t*, *J*_1_ = 7.5 and *J*_2_ = <1 Hz, H-10), 6.87 (1H, *d*, *J* = 7.5 Hz, H-12), 5.72 (1H, *q*, *J* = 7.0 Hz, H-19), 4.71 (1H, *sbr*, H-3), 4.37 (1H, *d*, *J* = 15.0 Hz, H_a_-21), 4.11 (1H, *sbr*, H-15), 4.02 (1H, *m*, H_a_-5), 3.84 (3H, *s*, CH_3_-22), 3.37 (1H, *d*, *J* = 15.0 Hz, H_b_-21), 3.31 (1H, *dd*, *J*_1_ = 12.0 and *J*_2_ = 6.5 Hz, H_b_-5), 2.68 (1H, *dt*, *J*_1_ = 13.5 and *J*_2_ = 6.7 Hz, H_a_-6), 2.59 (1H, *J*_1_ = 15.0 and *J*_2_ = 3.0 Hz, H_b_-14), 2.19 (1H, *dd*, *J*_1_ = 13.0 and *J*_2_ = 6.0 Hz, H_b_-6), 1.71 (3H, *d*, *J* = 7.0 Hz, CH_3_-18), 1.51 (1H, *dt*, *J*_1_ = 15.0 and *J*_2_ = 3.0 Hz, H_a_-14).

^13^C NMR (75 MHz, CDCl_3_), calibrated with solvent signal at δ 77.26 p.p.m., δ (p.p.m.): 167.2 (C-17), 164.0 (C-2), 143.1 (C-13), 133.2 (C-8), 130.9 (C-20), 129.8 (C-19), 129.6 (C-11), 122.3 (C-10), 121.4 (C-9), 110.5 (C-12), 102.3 (C-16), 61.5 (C-3), 55.1 (C-21), 55.0 (C-7), 54.1 (C-5), 51.8 (C-22), 43.2 (C-6), 29.3 (C-14), 28.5 (C-15), 13.8 (C-18).

## Refinement   

Crystal data, data collection and structure refinement details are summarized in Table 3 ▸. N-bound H atoms were refined freely. Methyls were refined as idealized rigid groups, with C—H = 0.98 Å and H—C—H = 109.5°. Other H atoms were included using a riding model starting from calculated positions, with aromatic C—H = 0.95 Å, methyl­ene C—H = 0.99 Å and methine C—H = 1.00 Å, with *U*_iso_(H) = 1.5*U*_eq_(C) for methyl H atoms and 1.2*U*_eq_(C) for other H atoms. The Flack parameter of 0.10 (13) is adequate to determine the absolute configuration.

## Supplementary Material

Crystal structure: contains datablock(s) I, global. DOI: 10.1107/S2056989017014529/dx2001sup1.cif

Structure factors: contains datablock(s) I. DOI: 10.1107/S2056989017014529/dx2001Isup2.hkl

CCDC reference: 1578796

Additional supporting information:  crystallographic information; 3D view; checkCIF report

## Figures and Tables

**Table 1:** Hydrogen-bond geometry (Å, °)

*D*—H⋯*A*	*D*—H	H⋯*A*	*D*⋯*A*	*D*—H⋯*A*
N1—H01⋯O1	0.90 (4)	2.20 (3)	2.754 (3)	119 (3)
N1—H01⋯O1′	0.90 (4)	2.23 (4)	3.018 (3)	146 (3)
N1′—H01′⋯O1′	0.89 (4)	2.20 (4)	2.745 (4)	119 (3)
N1′—H01′⋯O1	0.89 (4)	2.31 (4)	3.098 (3)	147 (4)
C18—H18*A*⋯O2	0.98	2.64	3.609 (4)	168
C18′—H18*D*⋯O2′	0.98	2.50	3.469 (4)	173

**Table 2:** Selected torsion angles (°)

C14—C3—N4—C21	−24.3 (3)	C14′—C3′—N4′—C21′	−24.0 (3)
C14—C3—C7—C2	−0.2 (4)	C14′—C3′—C7′—C2′	−0.1 (3)
C7—C2—C16—C15	−7.1 (4)	C7′—C2′—C16′—C15′	−9.0 (4)
C14—C15—C20—C21	−3.6 (3)	C14′—C15′—C20′—C21′	−1.6 (4)

**Table 3:** Experimental details

Crystal data	
Chemical formula	C_20_H_22_N_2_O_2_
*M*_r_	322.39
Crystal system, space group	Triclinic, *P*1
Temperature (K)	100
*a*, *b*, *c* (Å)	7.4750 (7), 7.7067 (6), 14.6585 (9)
α, β, γ (°)	104.696 (6), 92.637 (6), 94.548 (7)
*V* (Å^3^)	812.33 (11)
*Z*	2
Radiation type	Cu *K*α
μ (mm^−1^)	0.68
Crystal size (mm)	0.15 × 0.03 × 0.03

Data collection	
Diffractometer	Oxford Diffraction Xcalibur Atlas Nova
Absorption correction	Multi-scan (*CrysAlis PRO*; Rigaku Oxford Diffraction, 2015 ▸)
*T*_min_, *T*_max_	0.850, 1.000
No. of measured, independent and observed [*I* > 2σ(*I*)] reflections	40686, 6159, 5741
*R*_int_	0.065
(sin θ/λ)_max_ (Å^−1^)	0.630

Refinement	
*R*[*F* ^2^ > 2σ(*F* ^2^)], *wR*(*F* ^2^), *S*	0.041, 0.101, 1.04
No. of reflections	6159
No. of parameters	445
No. of restraints	3
H-atom treatment	H atoms treated by a mixture of independent and constrained refinement
Δρ_max_, Δρ_min_ (e Å^−3^)	0.21, −0.20
Absolute structure	Flack *x* parameter determined using 2335 quotients [(*I* ^+^) − (*I* ^−^)]/[(*I* ^+^) + (*I* ^−^)] (Parsons *et al.*, 2013 ▸)
Absolute structure parameter	0.10 (13)

Computer programs: *CrysAlis PRO* (Rigaku Oxford Diffraction, 2015 ▸), *SHELXS97* (Sheldrick, 2015 ▸), *SHELXL2017* (Sheldrick, 2015 ▸), *XP* (Siemens, 1994 ▸) and *SHELXL97* (Sheldrick, 2008 ▸).

## supplementary crystallographic information

### Crystal data

**Table:**

C_20_H_22_N_2_O_2_	*Z* = 2
*M**_r_* = 322.39	*F*(000) = 344
Triclinic, *P*1	*D*_x_ = 1.318 Mg m^−^^3^
*a* = 7.4750 (7) Å	Cu *K*α radiation, λ = 1.54184 Å
*b* = 7.7067 (6) Å	Cell parameters from 13753 reflections
*c* = 14.6585 (9) Å	θ = 5.9–75.8°
α = 104.696 (6)°	µ = 0.68 mm^−^^1^
β = 92.637 (6)°	*T* = 100 K
γ = 94.548 (7)°	Needle, colourless
*V* = 812.33 (11) Å^3^	0.15 × 0.03 × 0.03 mm

### Data collection

**Table:**

Oxford Diffraction Xcalibur Atlas Nova diffractometer	6159 independent reflections
Radiation source: micro-focus sealed X-ray tube	5741 reflections with *I* > 2σ(*I*)
Detector resolution: 10.3543 pixels mm^-1^	*R*_int_ = 0.065
ω scans	θ_max_ = 76.3°, θ_min_ = 6.0°
Absorption correction: multi-scan (CrysAlis PRO; Rigaku Oxford Diffraction, 2015)	*h* = −8→9
*T*_min_ = 0.850, *T*_max_ = 1.000	*k* = −9→9
40686 measured reflections	*l* = −18→18

### Refinement

**Table:**

Refinement on *F*^2^	Secondary atom site location: difference Fourier map
Least-squares matrix: full	Hydrogen site location: mixed
*R*[*F*^2^ > 2σ(*F*^2^)] = 0.041	H atoms treated by a mixture of independent and constrained refinement
*wR*(*F*^2^) = 0.101	*w* = 1/[σ^2^(*F*_o_^2^) + (0.0546*P*)^2^ + 0.0943*P*] where *P* = (*F*_o_^2^ + 2*F*_c_^2^)/3
*S* = 1.04	(Δ/σ)_max_ = 0.003
6159 reflections	Δρ_max_ = 0.21 e Å^−^^3^
445 parameters	Δρ_min_ = −0.20 e Å^−^^3^
3 restraints	Absolute structure: Flack *x* determined using 2335 quotients [(I+)-(I-)]/[(I+)+(I-)] (Parsons *et al.*, 2013)
Primary atom site location: structure-invariant direct methods	Absolute structure parameter: 0.10 (13)

### Special details

**Table:**

Geometry. All esds (except the esd in the dihedral angle between two l.s. planes) are estimated using the full covariance matrix. The cell esds are taken into account individually in the estimation of esds in distances, angles and torsion angles; correlations between esds in cell parameters are only used when they are defined by crystal symmetry. An approximate (isotropic) treatment of cell esds is used for estimating esds involving l.s. planes.

### Fractional atomic coordinates and isotropic or equivalent isotropic displacement parameters (Å^2^)

**Table:**

	*x*	*y*	*z*	*U*_iso_*/*U*_eq_	
N1	0.5190 (4)	0.4782 (3)	0.28497 (16)	0.0275 (5)	
H01	0.565 (5)	0.531 (4)	0.344 (2)	0.024 (8)*	
C2	0.4136 (4)	0.5639 (4)	0.23483 (18)	0.0256 (6)	
C3	0.3872 (4)	0.5708 (3)	0.05848 (18)	0.0265 (6)	
H3	0.488908	0.544687	0.016586	0.032*	
N4	0.2147 (4)	0.5149 (3)	0.00030 (15)	0.0284 (5)	
C5	0.1620 (5)	0.3308 (4)	0.00788 (18)	0.0297 (6)	
H5A	0.227379	0.242121	−0.036095	0.036*	
H5B	0.031164	0.299501	−0.007413	0.036*	
C6	0.2121 (5)	0.3316 (4)	0.11087 (18)	0.0286 (6)	
H6A	0.227532	0.208532	0.116945	0.034*	
H6B	0.120091	0.384084	0.153325	0.034*	
C7	0.3933 (4)	0.4526 (3)	0.13233 (17)	0.0261 (6)	
C8	0.5463 (4)	0.3352 (4)	0.12953 (19)	0.0275 (6)	
C9	0.6187 (5)	0.2186 (4)	0.0552 (2)	0.0306 (6)	
H9	0.577882	0.209391	−0.008380	0.037*	
C10	0.7526 (5)	0.1152 (4)	0.0754 (2)	0.0342 (7)	
H10	0.804766	0.036093	0.025129	0.041*	
C11	0.8104 (5)	0.1269 (4)	0.1685 (2)	0.0340 (7)	
H11	0.901397	0.054974	0.181004	0.041*	
C12	0.7372 (5)	0.2422 (4)	0.2439 (2)	0.0313 (6)	
H12	0.774867	0.248319	0.307545	0.038*	
C13	0.6079 (4)	0.3472 (3)	0.22279 (19)	0.0273 (6)	
C14	0.4024 (4)	0.7722 (3)	0.10664 (17)	0.0276 (6)	
H14A	0.374271	0.840809	0.059975	0.033*	
H14B	0.526261	0.813290	0.134814	0.033*	
C15	0.2670 (4)	0.8033 (3)	0.18458 (17)	0.0262 (6)	
H15	0.261568	0.935890	0.210291	0.031*	
C16	0.3457 (4)	0.7262 (4)	0.26288 (17)	0.0259 (6)	
C17	0.3919 (4)	0.8359 (4)	0.35900 (18)	0.0272 (6)	
C18	−0.0906 (5)	0.7944 (4)	0.2884 (2)	0.0329 (7)	
H18A	0.020729	0.866877	0.317662	0.049*	
H18B	−0.116899	0.698314	0.320004	0.049*	
H18C	−0.189989	0.871280	0.294681	0.049*	
C19	−0.0685 (5)	0.7125 (4)	0.18548 (19)	0.0298 (6)	
H19	−0.173052	0.650022	0.148756	0.036*	
C20	0.0805 (4)	0.7177 (4)	0.14015 (18)	0.0279 (6)	
C21	0.0755 (5)	0.6384 (4)	0.03389 (19)	0.0310 (6)	
H21A	−0.044282	0.572139	0.012555	0.037*	
H21B	0.088024	0.738777	0.003153	0.037*	
C22	0.4099 (6)	1.1314 (4)	0.4623 (2)	0.0376 (8)	
H22A	0.307137	1.145267	0.501813	0.056*	
H22B	0.453896	1.248929	0.453562	0.056*	
H22C	0.506223	1.082961	0.493159	0.056*	
O1	0.4602 (3)	0.7814 (3)	0.42280 (13)	0.0304 (5)	
O2	0.3547 (3)	1.0088 (3)	0.37120 (13)	0.0334 (5)	
N1'	0.5324 (4)	0.6183 (3)	0.59211 (16)	0.0282 (5)	
H01'	0.557 (6)	0.652 (6)	0.540 (3)	0.043 (10)*	
C2'	0.6443 (4)	0.5214 (4)	0.63243 (17)	0.0264 (6)	
C3'	0.7304 (5)	0.5197 (4)	0.80590 (18)	0.0294 (6)	
H3'	0.722276	0.626475	0.860512	0.035*	
N4'	0.6967 (4)	0.3522 (3)	0.83775 (16)	0.0308 (6)	
C5'	0.5046 (5)	0.2993 (4)	0.81692 (19)	0.0316 (7)	
H5'1	0.434494	0.372964	0.865486	0.038*	
H5'2	0.476610	0.170664	0.815071	0.038*	
C6'	0.4603 (5)	0.3321 (3)	0.71971 (18)	0.0281 (6)	
H6'1	0.330274	0.343124	0.709390	0.034*	
H6'2	0.497434	0.234551	0.668036	0.034*	
C7'	0.5744 (4)	0.5140 (3)	0.72739 (17)	0.0266 (6)	
C8'	0.4560 (5)	0.6659 (4)	0.74493 (19)	0.0289 (6)	
C9'	0.3666 (5)	0.7458 (4)	0.8232 (2)	0.0315 (6)	
H9'	0.384587	0.712996	0.880973	0.038*	
C10'	0.2503 (5)	0.8744 (4)	0.8161 (2)	0.0338 (7)	
H10'	0.190798	0.932032	0.869901	0.041*	
C11'	0.2201 (5)	0.9196 (4)	0.7310 (2)	0.0337 (7)	
H11'	0.138024	1.005923	0.727152	0.040*	
C12'	0.3084 (5)	0.8402 (4)	0.6510 (2)	0.0323 (7)	
H12'	0.288283	0.871058	0.592867	0.039*	
C13'	0.4263 (4)	0.7147 (4)	0.65995 (19)	0.0284 (6)	
C14'	0.9146 (5)	0.5310 (4)	0.7686 (2)	0.0314 (7)	
H14C	1.006195	0.509517	0.814476	0.038*	
H14D	0.944801	0.652462	0.759263	0.038*	
C15'	0.9137 (4)	0.3864 (4)	0.67316 (19)	0.0287 (6)	
H15'	1.039142	0.381604	0.652387	0.034*	
C16'	0.7971 (4)	0.4503 (4)	0.60213 (18)	0.0267 (6)	
C17'	0.8636 (5)	0.4777 (4)	0.51448 (18)	0.0290 (6)	
C18'	0.8904 (5)	0.0130 (4)	0.5238 (2)	0.0371 (7)	
H18D	0.932626	0.127975	0.511469	0.056*	
H18E	0.786806	−0.043359	0.479919	0.056*	
H18F	0.987267	−0.067022	0.514863	0.056*	
C19'	0.8357 (5)	0.0456 (4)	0.6238 (2)	0.0331 (7)	
H19'	0.787163	−0.056761	0.642494	0.040*	
C20'	0.8480 (5)	0.2031 (4)	0.68892 (19)	0.0294 (6)	
C21'	0.8080 (5)	0.2099 (4)	0.79049 (19)	0.0327 (7)	
H21C	0.746707	0.091838	0.791562	0.039*	
H21D	0.923995	0.225433	0.828359	0.039*	
C22'	1.1154 (5)	0.4795 (5)	0.4215 (2)	0.0430 (8)	
H22D	1.062668	0.393287	0.363550	0.065*	
H22E	1.245390	0.470793	0.427088	0.065*	
H22F	1.093033	0.601895	0.419177	0.065*	
O1'	0.7762 (3)	0.5340 (3)	0.45692 (14)	0.0325 (5)	
O2'	1.0348 (3)	0.4389 (3)	0.50224 (15)	0.0348 (5)	

### Atomic displacement parameters (Å^2^)

**Table:**

	*U*^11^	*U*^22^	*U*^33^	*U*^12^	*U*^13^	*U*^23^
N1	0.0277 (15)	0.0282 (10)	0.0254 (10)	−0.0005 (10)	−0.0019 (10)	0.0062 (8)
C2	0.0238 (17)	0.0274 (11)	0.0246 (11)	−0.0046 (11)	0.0011 (11)	0.0075 (9)
C3	0.0259 (17)	0.0288 (12)	0.0235 (11)	−0.0018 (11)	0.0014 (11)	0.0056 (9)
N4	0.0274 (16)	0.0313 (11)	0.0238 (10)	−0.0026 (10)	−0.0008 (10)	0.0042 (8)
C5	0.0274 (18)	0.0319 (13)	0.0255 (12)	−0.0055 (12)	−0.0017 (11)	0.0023 (10)
C6	0.0289 (18)	0.0291 (12)	0.0256 (12)	−0.0034 (12)	0.0020 (11)	0.0048 (9)
C7	0.0256 (18)	0.0267 (12)	0.0240 (11)	−0.0031 (11)	0.0009 (11)	0.0044 (9)
C8	0.0259 (18)	0.0268 (11)	0.0288 (12)	−0.0035 (12)	0.0030 (11)	0.0071 (9)
C9	0.0309 (19)	0.0294 (12)	0.0306 (12)	−0.0021 (12)	0.0053 (12)	0.0070 (10)
C10	0.033 (2)	0.0281 (12)	0.0412 (15)	−0.0001 (13)	0.0105 (14)	0.0077 (11)
C11	0.0252 (18)	0.0300 (12)	0.0483 (16)	0.0007 (12)	0.0044 (14)	0.0131 (11)
C12	0.0288 (19)	0.0298 (13)	0.0357 (13)	−0.0009 (12)	0.0026 (12)	0.0104 (10)
C13	0.0238 (17)	0.0250 (11)	0.0324 (13)	−0.0037 (11)	0.0029 (12)	0.0076 (10)
C14	0.0300 (18)	0.0279 (12)	0.0242 (11)	−0.0026 (12)	0.0010 (11)	0.0073 (9)
C15	0.0274 (18)	0.0271 (12)	0.0228 (11)	0.0006 (11)	−0.0001 (11)	0.0052 (9)
C16	0.0233 (17)	0.0285 (11)	0.0242 (12)	−0.0041 (11)	−0.0005 (11)	0.0058 (9)
C17	0.0251 (17)	0.0302 (12)	0.0241 (11)	−0.0032 (11)	0.0017 (11)	0.0047 (9)
C18	0.0303 (19)	0.0381 (14)	0.0320 (13)	0.0026 (13)	0.0037 (12)	0.0118 (11)
C19	0.0273 (18)	0.0312 (12)	0.0304 (12)	0.0003 (12)	−0.0010 (12)	0.0084 (10)
C20	0.0277 (18)	0.0297 (12)	0.0249 (12)	0.0030 (12)	−0.0012 (11)	0.0051 (9)
C21	0.0288 (18)	0.0363 (14)	0.0258 (12)	0.0006 (13)	−0.0029 (11)	0.0055 (10)
C22	0.045 (2)	0.0311 (13)	0.0293 (13)	−0.0006 (14)	−0.0064 (13)	−0.0030 (10)
O1	0.0303 (13)	0.0346 (9)	0.0244 (8)	−0.0005 (9)	−0.0025 (8)	0.0062 (7)
O2	0.0410 (15)	0.0293 (9)	0.0261 (9)	0.0021 (9)	−0.0053 (9)	0.0015 (7)
N1'	0.0248 (15)	0.0347 (11)	0.0245 (10)	−0.0011 (10)	0.0000 (10)	0.0081 (9)
C2'	0.0259 (17)	0.0291 (12)	0.0223 (11)	−0.0072 (11)	−0.0020 (11)	0.0065 (9)
C3'	0.0326 (19)	0.0302 (12)	0.0214 (11)	−0.0070 (12)	−0.0051 (11)	0.0035 (9)
N4'	0.0333 (16)	0.0327 (11)	0.0250 (10)	−0.0047 (11)	−0.0010 (10)	0.0079 (8)
C5'	0.035 (2)	0.0307 (12)	0.0264 (12)	−0.0072 (12)	0.0017 (12)	0.0061 (9)
C6'	0.0265 (18)	0.0293 (12)	0.0258 (12)	−0.0053 (12)	−0.0003 (11)	0.0050 (9)
C7'	0.0240 (17)	0.0309 (12)	0.0227 (11)	−0.0057 (12)	−0.0001 (11)	0.0057 (9)
C8'	0.0255 (17)	0.0297 (12)	0.0275 (12)	−0.0062 (12)	−0.0004 (11)	0.0030 (9)
C9'	0.0304 (19)	0.0310 (12)	0.0291 (12)	−0.0076 (12)	0.0035 (12)	0.0038 (10)
C10'	0.031 (2)	0.0281 (12)	0.0366 (13)	−0.0061 (13)	0.0065 (13)	0.0002 (10)
C11'	0.0262 (18)	0.0276 (12)	0.0439 (15)	−0.0029 (12)	0.0013 (13)	0.0049 (11)
C12'	0.0292 (19)	0.0318 (13)	0.0347 (13)	−0.0031 (12)	−0.0019 (12)	0.0086 (10)
C13'	0.0236 (17)	0.0293 (12)	0.0290 (12)	−0.0053 (12)	−0.0006 (11)	0.0042 (10)
C14'	0.0296 (19)	0.0345 (13)	0.0276 (12)	−0.0079 (12)	−0.0075 (12)	0.0085 (10)
C15'	0.0231 (17)	0.0363 (13)	0.0262 (12)	−0.0033 (12)	−0.0013 (11)	0.0095 (10)
C16'	0.0226 (16)	0.0311 (12)	0.0252 (12)	−0.0040 (11)	−0.0010 (11)	0.0075 (9)
C17'	0.0259 (18)	0.0338 (13)	0.0262 (12)	−0.0028 (12)	0.0007 (11)	0.0078 (10)
C18'	0.032 (2)	0.0423 (15)	0.0335 (14)	0.0024 (14)	0.0013 (13)	0.0044 (11)
C19'	0.0296 (19)	0.0366 (14)	0.0325 (13)	−0.0007 (13)	−0.0022 (12)	0.0094 (11)
C20'	0.0261 (18)	0.0348 (13)	0.0277 (12)	0.0002 (12)	−0.0021 (11)	0.0101 (10)
C21'	0.036 (2)	0.0355 (13)	0.0271 (12)	0.0000 (13)	−0.0012 (12)	0.0105 (10)
C22'	0.029 (2)	0.066 (2)	0.0410 (16)	0.0015 (17)	0.0098 (14)	0.0256 (15)
O1'	0.0279 (13)	0.0443 (11)	0.0276 (9)	0.0028 (10)	0.0022 (9)	0.0140 (8)
O2'	0.0241 (13)	0.0502 (12)	0.0344 (10)	0.0019 (10)	0.0047 (9)	0.0185 (9)

### Geometric parameters (Å, º)

**Table:**

N1—C2	1.368 (4)	N1'—C2'	1.373 (4)
N1—C13	1.409 (4)	N1'—C13'	1.401 (4)
N1—H01	0.90 (4)	N1'—H01'	0.89 (4)
C2—C16	1.360 (4)	C2'—C16'	1.351 (5)
C2—C7	1.523 (3)	C2'—C7'	1.522 (4)
C3—N4	1.483 (4)	C3'—N4'	1.488 (4)
C3—C14	1.526 (3)	C3'—C14'	1.509 (5)
C3—C7	1.583 (3)	C3'—C7'	1.590 (4)
C3—H3	1.0000	C3'—H3'	1.0000
N4—C5	1.475 (4)	N4'—C5'	1.458 (5)
N4—C21	1.483 (4)	N4'—C21'	1.479 (4)
C5—C6	1.537 (4)	C5'—C6'	1.535 (4)
C5—H5A	0.9900	C5'—H5'1	0.9900
C5—H5B	0.9900	C5'—H5'2	0.9900
C6—C7	1.554 (4)	C6'—C7'	1.558 (4)
C6—H6A	0.9900	C6'—H6'1	0.9900
C6—H6B	0.9900	C6'—H6'2	0.9900
C7—C8	1.509 (4)	C7'—C8'	1.501 (4)
C8—C9	1.387 (4)	C8'—C9'	1.385 (4)
C8—C13	1.400 (4)	C8'—C13'	1.403 (4)
C9—C10	1.394 (5)	C9'—C10'	1.389 (5)
C9—H9	0.9500	C9'—H9'	0.9500
C10—C11	1.392 (4)	C10'—C11'	1.391 (5)
C10—H10	0.9500	C10'—H10'	0.9500
C11—C12	1.395 (4)	C11'—C12'	1.399 (5)
C11—H11	0.9500	C11'—H11'	0.9500
C12—C13	1.382 (4)	C12'—C13'	1.385 (5)
C12—H12	0.9500	C12'—H12'	0.9500
C14—C15	1.547 (4)	C14'—C15'	1.551 (4)
C14—H14A	0.9900	C14'—H14C	0.9900
C14—H14B	0.9900	C14'—H14D	0.9900
C15—C20	1.535 (4)	C15'—C16'	1.526 (4)
C15—C16	1.535 (4)	C15'—C20'	1.535 (4)
C15—H15	1.0000	C15'—H15'	1.0000
C16—C17	1.457 (3)	C16'—C17'	1.457 (4)
C17—O1	1.225 (3)	C17'—O1'	1.228 (4)
C17—O2	1.352 (3)	C17'—O2'	1.346 (4)
C18—C19	1.503 (4)	C18'—C19'	1.504 (4)
C18—H18A	0.9800	C18'—H18D	0.9800
C18—H18B	0.9800	C18'—H18E	0.9800
C18—H18C	0.9800	C18'—H18F	0.9800
C19—C20	1.326 (5)	C19'—C20'	1.334 (4)
C19—H19	0.9500	C19'—H19'	0.9500
C20—C21	1.520 (3)	C20'—C21'	1.521 (4)
C21—H21A	0.9900	C21'—H21C	0.9900
C21—H21B	0.9900	C21'—H21D	0.9900
C22—O2	1.447 (3)	C22'—O2'	1.444 (4)
C22—H22A	0.9800	C22'—H22D	0.9800
C22—H22B	0.9800	C22'—H22E	0.9800
C22—H22C	0.9800	C22'—H22F	0.9800
			
C2—N1—C13	110.1 (2)	C2'—N1'—C13'	110.3 (2)
C2—N1—H01	122 (2)	C2'—N1'—H01'	122 (3)
C13—N1—H01	122 (2)	C13'—N1'—H01'	122 (3)
C16—C2—N1	129.7 (2)	C16'—C2'—N1'	129.9 (3)
C16—C2—C7	122.0 (2)	C16'—C2'—C7'	122.3 (3)
N1—C2—C7	108.0 (2)	N1'—C2'—C7'	107.7 (3)
N4—C3—C14	110.6 (2)	N4'—C3'—C14'	110.8 (3)
N4—C3—C7	107.2 (2)	N4'—C3'—C7'	106.4 (2)
C14—C3—C7	112.2 (2)	C14'—C3'—C7'	112.1 (2)
N4—C3—H3	108.9	N4'—C3'—H3'	109.1
C14—C3—H3	108.9	C14'—C3'—H3'	109.1
C7—C3—H3	108.9	C7'—C3'—H3'	109.1
C5—N4—C21	111.7 (3)	C5'—N4'—C21'	112.5 (2)
C5—N4—C3	104.9 (2)	C5'—N4'—C3'	105.1 (2)
C21—N4—C3	111.9 (2)	C21'—N4'—C3'	112.0 (2)
N4—C5—C6	105.9 (2)	N4'—C5'—C6'	105.5 (2)
N4—C5—H5A	110.5	N4'—C5'—H5'1	110.6
C6—C5—H5A	110.5	C6'—C5'—H5'1	110.6
N4—C5—H5B	110.5	N4'—C5'—H5'2	110.6
C6—C5—H5B	110.5	C6'—C5'—H5'2	110.6
H5A—C5—H5B	108.7	H5'1—C5'—H5'2	108.8
C5—C6—C7	102.1 (2)	C5'—C6'—C7'	101.8 (2)
C5—C6—H6A	111.3	C5'—C6'—H6'1	111.4
C7—C6—H6A	111.3	C7'—C6'—H6'1	111.4
C5—C6—H6B	111.3	C5'—C6'—H6'2	111.4
C7—C6—H6B	111.3	C7'—C6'—H6'2	111.4
H6A—C6—H6B	109.2	H6'1—C6'—H6'2	109.3
C8—C7—C2	101.4 (2)	C8'—C7'—C2'	101.4 (2)
C8—C7—C6	109.2 (2)	C8'—C7'—C6'	110.1 (3)
C2—C7—C6	111.2 (2)	C2'—C7'—C6'	111.1 (2)
C8—C7—C3	117.4 (2)	C8'—C7'—C3'	117.5 (2)
C2—C7—C3	113.5 (2)	C2'—C7'—C3'	113.2 (3)
C6—C7—C3	104.2 (2)	C6'—C7'—C3'	103.8 (2)
C9—C8—C13	120.0 (3)	C9'—C8'—C13'	119.6 (3)
C9—C8—C7	131.8 (3)	C9'—C8'—C7'	131.8 (3)
C13—C8—C7	108.0 (2)	C13'—C8'—C7'	108.3 (2)
C8—C9—C10	118.8 (3)	C8'—C9'—C10'	119.2 (3)
C8—C9—H9	120.6	C8'—C9'—H9'	120.4
C10—C9—H9	120.6	C10'—C9'—H9'	120.4
C11—C10—C9	120.6 (3)	C9'—C10'—C11'	120.6 (3)
C11—C10—H10	119.7	C9'—C10'—H10'	119.7
C9—C10—H10	119.7	C11'—C10'—H10'	119.7
C10—C11—C12	121.2 (3)	C10'—C11'—C12'	121.2 (3)
C10—C11—H11	119.4	C10'—C11'—H11'	119.4
C12—C11—H11	119.4	C12'—C11'—H11'	119.4
C13—C12—C11	117.6 (3)	C13'—C12'—C11'	117.3 (3)
C13—C12—H12	121.2	C13'—C12'—H12'	121.3
C11—C12—H12	121.2	C11'—C12'—H12'	121.3
C12—C13—C8	121.9 (3)	C12'—C13'—N1'	128.8 (3)
C12—C13—N1	128.8 (3)	C12'—C13'—C8'	122.1 (3)
C8—C13—N1	109.3 (2)	N1'—C13'—C8'	109.1 (3)
C3—C14—C15	107.8 (2)	C3'—C14'—C15'	108.5 (2)
C3—C14—H14A	110.1	C3'—C14'—H14C	110.0
C15—C14—H14A	110.1	C15'—C14'—H14C	110.0
C3—C14—H14B	110.1	C3'—C14'—H14D	110.0
C15—C14—H14B	110.1	C15'—C14'—H14D	110.0
H14A—C14—H14B	108.5	H14C—C14'—H14D	108.4
C20—C15—C16	115.8 (2)	C16'—C15'—C20'	115.4 (2)
C20—C15—C14	108.6 (2)	C16'—C15'—C14'	106.2 (2)
C16—C15—C14	105.6 (2)	C20'—C15'—C14'	108.2 (2)
C20—C15—H15	108.9	C16'—C15'—H15'	109.0
C16—C15—H15	108.9	C20'—C15'—H15'	109.0
C14—C15—H15	108.9	C14'—C15'—H15'	109.0
C2—C16—C17	118.7 (3)	C2'—C16'—C17'	118.8 (3)
C2—C16—C15	116.7 (2)	C2'—C16'—C15'	117.0 (2)
C17—C16—C15	122.6 (2)	C17'—C16'—C15'	122.4 (3)
O1—C17—O2	122.4 (2)	O1'—C17'—O2'	121.9 (3)
O1—C17—C16	124.7 (3)	O1'—C17'—C16'	124.5 (3)
O2—C17—C16	112.9 (2)	O2'—C17'—C16'	113.6 (2)
C19—C18—H18A	109.5	C19'—C18'—H18D	109.5
C19—C18—H18B	109.5	C19'—C18'—H18E	109.5
H18A—C18—H18B	109.5	H18D—C18'—H18E	109.5
C19—C18—H18C	109.5	C19'—C18'—H18F	109.5
H18A—C18—H18C	109.5	H18D—C18'—H18F	109.5
H18B—C18—H18C	109.5	H18E—C18'—H18F	109.5
C20—C19—C18	127.3 (3)	C20'—C19'—C18'	126.9 (3)
C20—C19—H19	116.3	C20'—C19'—H19'	116.6
C18—C19—H19	116.4	C18'—C19'—H19'	116.6
C19—C20—C21	120.2 (3)	C19'—C20'—C21'	120.2 (3)
C19—C20—C15	126.1 (2)	C19'—C20'—C15'	125.5 (3)
C21—C20—C15	113.7 (3)	C21'—C20'—C15'	114.1 (2)
N4—C21—C20	115.7 (2)	N4'—C21'—C20'	116.7 (2)
N4—C21—H21A	108.3	N4'—C21'—H21C	108.1
C20—C21—H21A	108.3	C20'—C21'—H21C	108.1
N4—C21—H21B	108.3	N4'—C21'—H21D	108.1
C20—C21—H21B	108.3	C20'—C21'—H21D	108.1
H21A—C21—H21B	107.4	H21C—C21'—H21D	107.3
O2—C22—H22A	109.5	O2'—C22'—H22D	109.5
O2—C22—H22B	109.5	O2'—C22'—H22E	109.5
H22A—C22—H22B	109.5	H22D—C22'—H22E	109.5
O2—C22—H22C	109.5	O2'—C22'—H22F	109.5
H22A—C22—H22C	109.5	H22D—C22'—H22F	109.5
H22B—C22—H22C	109.5	H22E—C22'—H22F	109.5
C17—O2—C22	116.7 (2)	C17'—O2'—C22'	116.7 (2)
			
C13—N1—C2—C16	−159.8 (3)	C13'—N1'—C2'—C16'	−160.4 (3)
C13—N1—C2—C7	14.9 (3)	C13'—N1'—C2'—C7'	15.6 (3)
C14—C3—N4—C5	−145.6 (2)	C14'—C3'—N4'—C5'	−146.4 (2)
C7—C3—N4—C5	−23.1 (3)	C7'—C3'—N4'—C5'	−24.3 (3)
C14—C3—N4—C21	−24.3 (3)	C14'—C3'—N4'—C21'	−24.0 (3)
C7—C3—N4—C21	98.2 (3)	C7'—C3'—N4'—C21'	98.1 (3)
C21—N4—C5—C6	−82.6 (3)	C21'—N4'—C5'—C6'	−81.1 (3)
C3—N4—C5—C6	38.8 (3)	C3'—N4'—C5'—C6'	41.0 (3)
N4—C5—C6—C7	−38.5 (3)	N4'—C5'—C6'—C7'	−40.5 (3)
C16—C2—C7—C8	157.7 (3)	C16'—C2'—C7'—C8'	158.3 (3)
N1—C2—C7—C8	−17.5 (3)	N1'—C2'—C7'—C8'	−18.1 (3)
C16—C2—C7—C6	−86.3 (3)	C16'—C2'—C7'—C6'	−84.7 (3)
N1—C2—C7—C6	98.5 (3)	N1'—C2'—C7'—C6'	98.8 (3)
C16—C2—C7—C3	30.8 (4)	C16'—C2'—C7'—C3'	31.6 (4)
N1—C2—C7—C3	−144.4 (3)	N1'—C2'—C7'—C3'	−144.8 (2)
C5—C6—C7—C8	−103.4 (2)	C5'—C6'—C7'—C8'	−102.6 (3)
C5—C6—C7—C2	145.5 (2)	C5'—C6'—C7'—C2'	145.9 (3)
C5—C6—C7—C3	22.9 (3)	C5'—C6'—C7'—C3'	24.0 (3)
N4—C3—C7—C8	120.2 (3)	N4'—C3'—C7'—C8'	120.9 (3)
C14—C3—C7—C8	−118.2 (3)	C14'—C3'—C7'—C8'	−117.8 (3)
N4—C3—C7—C2	−121.8 (3)	N4'—C3'—C7'—C2'	−121.4 (2)
C14—C3—C7—C2	−0.2 (4)	C14'—C3'—C7'—C2'	−0.1 (3)
N4—C3—C7—C6	−0.7 (3)	N4'—C3'—C7'—C6'	−0.8 (3)
C14—C3—C7—C6	120.9 (3)	C14'—C3'—C7'—C6'	120.5 (2)
C2—C7—C8—C9	−170.8 (3)	C2'—C7'—C8'—C9'	−171.9 (3)
C6—C7—C8—C9	71.7 (4)	C6'—C7'—C8'—C9'	70.4 (4)
C3—C7—C8—C9	−46.6 (4)	C3'—C7'—C8'—C9'	−48.1 (5)
C2—C7—C8—C13	14.1 (3)	C2'—C7'—C8'—C13'	14.5 (3)
C6—C7—C8—C13	−103.3 (3)	C6'—C7'—C8'—C13'	−103.2 (3)
C3—C7—C8—C13	138.4 (2)	C3'—C7'—C8'—C13'	138.3 (3)
C13—C8—C9—C10	0.0 (4)	C13'—C8'—C9'—C10'	−0.6 (4)
C7—C8—C9—C10	−174.6 (3)	C7'—C8'—C9'—C10'	−173.6 (3)
C8—C9—C10—C11	1.0 (5)	C8'—C9'—C10'—C11'	1.6 (5)
C9—C10—C11—C12	−0.3 (5)	C9'—C10'—C11'—C12'	−1.4 (5)
C10—C11—C12—C13	−1.3 (5)	C10'—C11'—C12'—C13'	0.2 (5)
C11—C12—C13—C8	2.3 (4)	C11'—C12'—C13'—N1'	−179.0 (3)
C11—C12—C13—N1	−177.3 (3)	C11'—C12'—C13'—C8'	0.8 (5)
C9—C8—C13—C12	−1.6 (4)	C2'—N1'—C13'—C12'	173.8 (3)
C7—C8—C13—C12	174.1 (3)	C2'—N1'—C13'—C8'	−6.1 (3)
C9—C8—C13—N1	178.0 (3)	C9'—C8'—C13'—C12'	−0.6 (5)
C7—C8—C13—N1	−6.3 (3)	C7'—C8'—C13'—C12'	173.9 (3)
C2—N1—C13—C12	174.0 (3)	C9'—C8'—C13'—N1'	179.3 (3)
C2—N1—C13—C8	−5.6 (3)	C7'—C8'—C13'—N1'	−6.2 (3)
N4—C3—C14—C15	70.9 (3)	N4'—C3'—C14'—C15'	70.5 (3)
C7—C3—C14—C15	−48.7 (3)	C7'—C3'—C14'—C15'	−48.2 (3)
C3—C14—C15—C20	−53.1 (3)	C3'—C14'—C15'—C16'	70.4 (3)
C3—C14—C15—C16	71.7 (3)	C3'—C14'—C15'—C20'	−54.0 (3)
N1—C2—C16—C17	2.5 (5)	N1'—C2'—C16'—C17'	1.2 (4)
C7—C2—C16—C17	−171.5 (3)	C7'—C2'—C16'—C17'	−174.3 (2)
N1—C2—C16—C15	166.9 (3)	N1'—C2'—C16'—C15'	166.6 (3)
C7—C2—C16—C15	−7.1 (4)	C7'—C2'—C16'—C15'	−9.0 (4)
C20—C15—C16—C2	76.4 (4)	C20'—C15'—C16'—C2'	78.4 (3)
C14—C15—C16—C2	−43.8 (3)	C14'—C15'—C16'—C2'	−41.4 (3)
C20—C15—C16—C17	−119.9 (3)	C20'—C15'—C16'—C17'	−116.8 (3)
C14—C15—C16—C17	120.0 (3)	C14'—C15'—C16'—C17'	123.4 (3)
C2—C16—C17—O1	−14.1 (5)	C2'—C16'—C17'—O1'	−13.2 (4)
C15—C16—C17—O1	−177.6 (3)	C15'—C16'—C17'—O1'	−177.7 (3)
C2—C16—C17—O2	164.7 (3)	C2'—C16'—C17'—O2'	165.4 (3)
C15—C16—C17—O2	1.2 (4)	C15'—C16'—C17'—O2'	0.9 (4)
C18—C19—C20—C21	−175.7 (3)	C18'—C19'—C20'—C21'	−172.5 (3)
C18—C19—C20—C15	2.6 (5)	C18'—C19'—C20'—C15'	3.0 (6)
C16—C15—C20—C19	59.4 (4)	C16'—C15'—C20'—C19'	63.8 (4)
C14—C15—C20—C19	177.9 (3)	C14'—C15'—C20'—C19'	−177.4 (3)
C16—C15—C20—C21	−122.2 (3)	C16'—C15'—C20'—C21'	−120.4 (3)
C14—C15—C20—C21	−3.6 (3)	C14'—C15'—C20'—C21'	−1.6 (4)
C5—N4—C21—C20	82.5 (3)	C5'—N4'—C21'—C20'	84.4 (3)
C3—N4—C21—C20	−34.8 (3)	C3'—N4'—C21'—C20'	−33.8 (4)
C19—C20—C21—N4	−130.9 (3)	C19'—C20'—C21'—N4'	−136.0 (3)
C15—C20—C21—N4	50.6 (3)	C15'—C20'—C21'—N4'	48.0 (4)
O1—C17—O2—C22	3.8 (5)	O1'—C17'—O2'—C22'	4.5 (4)
C16—C17—O2—C22	−175.0 (3)	C16'—C17'—O2'—C22'	−174.2 (3)

### Hydrogen-bond geometry (Å, º)

**Table:**

*D*—H···*A*	*D*—H	H···*A*	*D*···*A*	*D*—H···*A*
N1—H01···O1	0.90 (4)	2.20 (3)	2.754 (3)	119 (3)
N1—H01···O1′	0.90 (4)	2.23 (4)	3.018 (3)	146 (3)
N1′—H01′···O1′	0.89 (4)	2.20 (4)	2.745 (4)	119 (3)
N1′—H01′···O1	0.89 (4)	2.31 (4)	3.098 (3)	147 (4)
C18—H18*A*···O2	0.98	2.64	3.609 (4)	168
C18′—H18*D*···O2′	0.98	2.50	3.469 (4)	173
